# A survey of Kansas beef producers and consumers who participate in business-to-consumer marketing of beef

**DOI:** 10.1093/tas/txad125

**Published:** 2023-11-04

**Authors:** Travis G O’Quinn, Katie R Lybarger, Gregory A Ibendahl, Yue Teng Vaughan, Junehee Kwon

**Affiliations:** Department of Animal Sciences and Industry, Kansas State University, Manhattan, KS 66506, USA; Department of Animal Sciences and Industry, Kansas State University, Manhattan, KS 66506, USA; Department of Agricultural Economics, Kansas State University, Manhattan, KS 66506, USA; Department of Hospitality Management, Kansas State University, Manhattan, KS 66506, USA; Department of Retail, Hospitality, and Tourism Management, University of Tennessee, Knoxville, TN 37996, USA

**Keywords:** beef, business-to-consumer, consumers, local, marketing, producers

## Abstract

Following the coronavirus disease 2019 (COVID-19) pandemic, producer and consumer interest in business-to-consumer (B2C) beef sales increased. The objective of the current study was to assess current B2C beef producer and consumer attitudes and understandings of the B2C beef marketing process in order to identify knowledge gaps and strategies to improve producer/consumer interactions. Both producers and customers of local beef were recruited using a large online platform (https://shopkansasfarms.com), and descriptive statistics were used to summarize the data. In total, 41 B2C beef producers and 174 consumers who had either previously participated in B2C marketing or intended to participate were surveyed. Most producers (69.8%) only produced beef and produced only a small number (1 to 20 head) of animals per year. Many (43.9%) reported selling 100% of beef directly to consumers, while 29.3% reported selling less than 20% through this channel. Almost all (97.3%) of the producers indicated that increased sales directly to consumers would be desirable, with most (87.1%) considering this marketing channel as the most profitable. Marketing beef in smaller portions, including portioned cuts, was popular, reported by more than 62% of producers, while whole carcass sales were lower. Word-of-mouth (91.3%) and social media (65.8%) were the most popular forms of advertisement used by producers and more than one-third of producers (38.9%) reported having trouble with customers regarding a sale. Over 60% of consumers indicated they had purchased B2C beef less than 5 times, with more than 73% indicating that more than 75% of their beef purchased was local. Low take-home weights, portion sizes, and quality were among consumers’ most cited troubles. Lack of freezer space (25%), price (24.9%), and quantity of product (41.7%) were reported as the largest barriers to consumer participation in B2C marketing. Both consumers and producers indicated that consumer testimonials would be the most beneficial in improving producer/consumer interactions, with educational materials from government sources viewed as the least beneficial. These results provide a baseline for B2C beef marketing and provide insight into impactful strategies to use to assist in this process.

## Teaser

Kansas beef producers and consumers who participate in business-to-consumer (B2C) beef marketing find value and opportunity in direct-to-consumer marketing of beef.

## Introduction

Business-to-consumer (**B2C**) marketing refers to businesses selling their products directly to end-product consumers as opposed to other businesses (Kumar and Raheja, 2012). From an agricultural products standpoint, this type of marketing is commonly found in farmers’ markets, on-farm stores, roadside stands, pick-your-own operations, and online marketplaces. Consumers’ interest in local foods is robust, with direct-marketed agricultural food product sales totaling over $9 billion nationally and over $166 million for the region consisting of Kansas, Iowa, Minnesota, Missouri, Nebraska, North Dakota, and South Dakota in 2020 (USDA-NASS, 2022). In the meat industry, such B2C marketing has traditionally been more limited due to the added challenges associated with animal harvest and the need for carcass processing and cold-chain management. Despite this, consumer and food service interests in and demand for direct-marketed beef have been growing (Telligman et al., 2017; McKay et al., 2019).

Consumer interest in direct-marketed beef products spiked in 2020 and the subsequent years following the coronavirus disease 2019 (COVID-19) pandemic (Langusch, 2021). Pandemic-related challenges facing the beef supply chain resulted in empty retail store shelves, higher beef prices, and a lack of the desired product mix nationally (Hobbs, 2021). For many consumers, these challenges increased their desire to seek out B2C beef suppliers. For many, this newfound desire was met with unique product-flow limitations. Though suppliers were available with cattle ready for harvest, consumers and beef producers were often faced with back-logged harvest schedules, with local processors often booked out for periods of close to 2 yr (Bir et al., 2021). This extra demand created additional burdens on small meat processors and highlighted an overall need for expanded numbers and processing capacity within this sector. In Kansas, the Kansas Department of Agriculture (**KDA**) licensed seven new custom meat processing and 5 new state-inspected facilities from 2020 through the spring of 2022, bringing the total number of small and very-small processing facilities to more than 100 across the state (KDA, 2023a, b), adding capacity to help meet growing demand and thus offering consumers greater opportunities for B2C beef.

In addition to increased slaughter capacity, additional consumer-focused channels were either created or grew in scope to reach consumers looking to participate in B2C marketing in the wake of the COVID-19 pandemic. One such online channel, Shop Kansas Farms supported by the Kansas Farm Bureau, worked to help pair producers of livestock and other agricultural goods with consumers looking for such local products (Kansas Farm Bureau, 2023). Additionally, state government-supported programs such as Kansas Local Foods (KSU, 2023) housed at Kansas State University and KDA’s Local Food and Farm Task Force (KDA, 2023c) worked to help expand, develop, and support the local food system in Kansas. Such resources have proven to be critical to help support local foods and the development of B2C marketing channels for agricultural products, in many cases providing an important link between consumers and agricultural producers.

Despite the increased demand for B2C beef products, little is known about the practices and needs of beef producers operating B2C businesses. Likewise, consumer knowledge and understanding of the B2C beef process and overall expectations are not well understood. Variation in producer practices coupled with consumer variation on expectations can create challenges for the B2C beef marketing model, potentially resulting in either a producer or consumer leaving the B2C market. It was therefore the objective of the current work to survey both beef producers and consumers who participate in B2C beef marketing in Kansas to assess their practices and knowledge level, and identify potential challenges faced by both, in order to provide valuable baseline information for consideration by those participating in B2C beef markets.

## Materials and Methods

Prior to data collection, the research protocol was reviewed and approved by the University Research Compliance Office at Kansas State University (IRB approval #10758). In addition, before participants accessed the survey questions, they were informed of the purpose, procedures, benefits, and risks of completing the survey. Only those who indicated their consent to participate in the research accessed the survey site and provided data.

### Participant Selection

The participants for this study were beef producers and customers with direct B2C beef purchasing experience in Kansas. Both groups were invited to complete the survey to share their experience, knowledge, and challenges toward B2C beef markets. The participants were recruited in two channels. First, the researchers identified 113 farmers and ranchers listed as sellers of beef at the Shop Kansas Farms website (https://shopkansasfarms.com/) and sent the survey invitation via email. In addition, the organizer of the Shop Kansas Farm group posted the invitation to the survey on their social networking site. The Shop Kansas Farm group was developed in the midst of COVID-19 to help farmers and ranchers sell meat and other agricultural products directly to consumers. Including more than 160,000 members, Shop Kansas Farms has become one the most trusted online platforms in providing a link for direct-to-consumer marketing for both farmers and consumers. Using these two recruitment protocols, a total of 43 beef producers and 174 consumers completed the survey and were included in the survey. It is recognized that using such a recruitment method may have not included consumers and producers who do not participate in the Shop Kansas Farms website; however, no method to identify and reach such producers and consumers existed and was available.

### Questionnaire Development, Data Collection, and Data Analysis

The questionnaire was developed based on the study objectives. Participants were asked to indicate their experience and their attitudes toward/satisfaction with B2C beef marketing experience. The survey only asked producers and consumers questions that directly related to their individual experiences; thus a varied number of participants were included for each question set. Data were collected online using Qualtrics, an online survey system. Descriptive statistics such as frequency, percentages, means, and standard deviation were used to summarize the data using SPSS (Version 27.0).

## Results and Discussion

### Beef Producer Survey

In total, 43 producers participated in the survey, with 41 indicating they sold beef (Table 1) and stating they had previously participated in B2C beef marketing. Of the beef producers surveyed, only 30.2% sold livestock species in addition to beef, with the majority (69.8%) selling only beef. Half (50%) of the surveyed producers indicated they sold between 1 and 20 total animals in 2020, followed by 15.8% who sold 21 to 40, with 21% selling more than 80 heads (Table 2). This indicates two populations of producers who participated in B2C beef sales—those who had small numbers of animals produced and sold primarily through B2C channels and those who had larger animal numbers and sold a smaller portion of animals through B2C channels. This was further supported by the reported customer basis for beef sales. Beef sales directly to individual customers represented 100% of cattle sold for 43.9% of producers surveyed, but another 22% of producers indicated only 1% to 20% of their animal sales were directly sold to individuals. This helps to support the idea of the 2 main populations of producers who sell into B2C markets.

**Table 1.** Summary of business-to-consumer (B2C) beef producers responses regarding food animal production (*N* = 43)

Characteristic	Response	Percentage of responses
Animal (*n* = 43)	Beef cattle	69.8
	Beef cattle, chicken/turkey	4.7
	Beef cattle, goat, chicken/turkey, other	2.3
	Beef cattle, pigs	9.3
	Beef cattle, pigs, chicken/turkey	2.3
	Beef cattle, pigs, goat, chicken/turkey	2.3
	Beef cattle, pigs, lamb	2.3
	Beef cattle, pigs, lamb, chicken/turkey	2.3
	Goat, lamb, chicken/turkey, other	2.3
	Lamb	2.3
Other responses (*n* = 2)	Duck	50.0
	Duck, quail	50.0

**Table 2.** Summary of responses related to beef sales practices from business-to-consumer (B2C) beef producers (*N* = 41)

Characteristic	Response	Percentage of responses
Head of finished beef cattle sold in 2020 (*n* = 38)	1 to 20	50.0
	21 to 40	15.8
	41 to 60	7.9
	61 to 80	0.0
	81 to 100	5.3
	101 to 200	10.5
	> 500	10.5
Estimate of percentage of cattle sold to individual consumers in 2020 (*n* = 41)	0	7.3
	1 to 20	22.0
	21 to 40	4.9
	41 to 60	7.3
	61 to 80	4.9
	81 to 99	9.8
	100	43.9
Estimate of percentage of cattle sold to foodservice in 2020 (*n* = 41)	0	92.7
	1 to 20	4.9
	21 to 40	2.4
	41 to 60	0.0
	61 to 80	0.0
	81 to 99	0.0
	100	0.0
Estimate of percentage of cattle sold to large beef processors in 2020^1^ (*n* = 41)	0	70.7
	1 to 20	4.9
	21 to 40	2.4
	41 to 60	0.0
	61 to 80	7.3
	81 to 99	14.6
	100	0.0
Estimate of percentage of cattle sold to supermarkets in 2020 (*n* = 41)	0	92.7
	1 to 20	7.3
	21 to 40	0.0
	41 to 60	0.0
	61 to 80	0.0
	81 to 99	0.0
	100	0.0
Estimate of percentage of cattle sold through other marketing channels in 2020 (*n* = 41)	0	82.9
	1 to 20	0.0
	21 to 40	0.0
	41 to 60	4.9
	61 to 80	4.9
	81 to 99	0.0
	100	7.3
Other responses^2^ (*n* = 7)	Live auction	71.4
	Raised for personal consumption	28.6

^1^Including Cargill, Tyson, National Beef, etc.

^2^Question appeared only to producers who responded an estimate of cattle sold through other market channels.

Additionally, sales to large beef packers represented 81% to 99% of sales for 14.6%, and 61% to 80% of sales for 7.3% of producers, with the majority (70.7%) reporting selling 0% of cattle to these companies (Table 2). It is also noteworthy that the overwhelming majority of surveyed beef producers reported not selling beef to supermarkets (92.7%) or to foodservice (92.7%). Whether these marketing channels are not available to, have potential limitations, or have just not been explored by these producers is unclear. Previous work has indicated selling local products through an intermediate such as a supermarket or restaurant results in 3 times the value compared to selling directly to consumers (Low and Vogel, 2011). Additionally, larger farms are more likely to sell through an intermediate, with close to 70% of large farms selling locally sourced foods through an intermediate, compared to less than 30% of small farms (Low and Vogel, 2011). Thus, selling products to local restaurants or supermarket chains may offer an overlooked opportunity for greater profitability for Kansas B2C beef producers. Other marketing channels including live auctions and beef raised for personal consumption were also practiced by the surveyed producers.

When asked about their perceptions regarding beef marketing channels, producer opinions varied based on channel (Table 3). When asked about the desirability of increased sales of beef directly to individuals, 97.3% of producers indicated such increases would be either somewhat or very desirable, with none indicating that increased B2C sales would be undesirable. The reverse trend was true when asked about sales to large beef processors. A total of 40.6% of producers indicated that increased sales of beef to large processors would be at least somewhat undesirable, with only 25% indicating it would be desirable. Though most producers indicated they did not sell directly to restaurants or foodservice operators, 62.5% indicated increased sales to these customers would be desirable. However, in another marketing channel where producers indicated low involvement, only 29% indicated increased sales to supermarkets would be desirable. This difference between perceptions related to foodservice and supermarkets is interesting, as the vast majority of producers indicated they did not sell beef to these customer bases, but yet had very different opinions on the desirability of such sales.

**Table 3.** Summary of responses from business-to-consumer (B2C) beef producers regarding desirability of increased beef sales to each customer group (*N* = 41)

Characteristic	Response	Percentage of responses
Increased individual consumer sales (*n* = 37)	Very desirable	73.0
	Somewhat desirable	24.3
	Neutral/undecided	2.7
	Somewhat undesirable	0.0
	Very undesirable	0.0
Increased foodservice sales (*n* = 32)	Very desirable	12.5
	Somewhat desirable	40.6
	Neutral/undecided	43.8
	Somewhat undesirable	0.0
	Very undesirable	3.1
Increased large beef processor sales (*n* = 32)	Very desirable	12.5
	Somewhat desirable	12.5
	Neutral/undecided	34.4
	Somewhat undesirable	12.5
	Very undesirable	28.1
Increased supermarket sales (*n* = 31)	Very desirable	12.9
	Somewhat desirable	16.1
	Neutral/undecided	61.3
	Somewhat undesirable	6.5
	Very undesirable	3.2
Increased other sales^1^ (*n* = 26)	Very desirable	19.2
	Somewhat desirable	19.2
	Neutral/undecided	57.7
	Somewhat undesirable	0.0
	Very undesirable	3.8

^1^Text responses can be found in Table 2

Some of these differences in opinions related to preferred marketing channels can be supported by the producers’ opinions related to the profitability of each (Table 4). Local branding of beef products has been shown to be advantageous, with local ground beef previously shown to generate premiums of $2.82/ kg (Stutzman, 2020) and resulting in improved consumer perceptions of eating quality (Harr et al., 2022). In the current study, when asked to rank the profitability of the various marketing channels, 87.1% of producers indicated sales directly to individual customers or B2C as the most profitable, with all producers ranking B2C marketing in the top 3 for profitability. Conversely, 74.2% of producers identified other channels (including live auctions and personal consumption) in the bottom 2 for profitability, with more than half (51.6%) identifying it as the least profitable. However, few producers (<20%) had indicated they sold beef through these channels. It is also noteworthy that 83.9% of producers indicated sales to restaurants in the top 3 for profitability, with 6.5% considering it the most profitable, highlighting the previously discussed perceptions related to desirability for increased sales through this channel. This belief is justified, as local food on menus drives consumer demand when sold at premium prices compared to selling local foods at the same price as commodity foods (Alfnes and Sharma, 2010) and are thus more profitable for restaurants.

**Table 4.** Summary of responses from business-to-consumer (B2C) beef producers who reported regarding ranking of profitability among each consumer group, with 1 being most profitable to 5 being least profitable (*N* = 41)

Characteristic	Response	Percentage of responses
Individual consumer sales (*n* = 31)	1	87.1
	2	9.7
	3	3.2
	4	0.0
	5	0.0
Foodservice sales (*n* = 31)	1	6.5
	2	35.5
	3	41.9
	4	12.9
	5	3.2
Large beef processor sales (*n* = 31)	1	3.2
	2	32.3
	3	19.4
	4	25.8
	5	19.4
Supermarket sales (*n* = 31)	1	0.0
	2	9.7
	3	25.8
	4	38.7
	5	25.8
Other sales^1^ (*n* = 31)	1	3.2
	2	12.9
	3	9.7
	4	22.6
	5	51.6

^1^Text responses can be found in Table 2

Sales to supermarkets were not viewed as profitable by producers, with 64.5% classifying such sales in the bottom 2 markets based on profitability. Again, sales to large beef processors seemed to divide producers, with 35.5% indicating sales through this channel in the top 2 for profitability, but 45.2% classifying it in the bottom 2 for the same trait. This is likely due to the large differences in beef marketing programs currently available to producers by large processors. Beef producers selling to large packers as part of an alternative marketing arrangement often are eligible to market cattle on quality-based premium grids or formula-based marketing in which animals that produce the most desirable, and thus profitable, traits are rewarded with higher premiums (Muth et al., 2008). However, in most cases, this requires beef producers to have sufficient animal numbers and consistent supply for negation of such marketing arrangements with packers. As reported, many of the producers who participated in the current survey had smaller numbers of heads, thus likely limiting their ability to negotiate with packers for the highest premiums and thus likely influencing their profitability in selling cattle through this channel.

Finally, although no reported producers in the current study indicated sales through farmers’ markets, farmers’ markets are a common form of B2C marketing of agricultural goods, including meat items. However, challenges related to cold-chain management of frozen meat items may make such marketing channels difficult for beef producers selling meat. Additionally, consumers have shown an unwillingness to pay premiums for food products sold at farmers’ markets (Printezis and Grebitus, 2018), thus providing some economically related justification to the surveyed producers avoiding this marketing channel.

Table 5 details producer responses to questions specifically regarding their sales of beef to individual customers in the B2C format. It is clear that the number of producers who sell beef directly to individual customers has increased over time, with 50% of the surveyed producers selling in the B2C format for less than 5 yr and only 22.3% selling beef directly to individual customers for more than 10 yr. Of sales, more than 55% of producers indicated that 75% or more of their sales were from returning customers, compared to only 13.9% of producers who reported no returning customers. The high level of repeat customers is not surprising, as consumers who participate in local food purchases often have a high level of trust in the quality of local beef and an established trust with local producers through the relationships such B2C marketing brings (Telligman et al., 2017).

**Table 5.** Summary of responses from business-to-consumer (B2C) beef producers regarding sales to individual consumers (*N* = 41)

Characteristic	Response	Percentage of responses
Estimate number of years selling to individual consumers (*n* = 36)	0	5.6
	1 to 5	44.4
	6 to 10	27.8
	11 to 15	2.8
	16 to 20	5.6
	>20	13.9
Percentage of customers being repeat customers (*n* = 36)	0	13.9
	25	22.2
	50	8.3
	75	47.2
	100	8.3
Form of sales (*n* = 35)	Half beef	5.7
	Half beef, portion cuts	2.9
	Half beef, quarter beef	8.6
	Portion cuts	5.7
	Portion cuts, butcher bag	2.9
	Whole beef	8.6
	Whole beef, half beef	8.6
	Whole beef, half beef, quarter beef	34.3
	Whole beef, half beef, quarter beef, portion cuts	14.3
	Whole beef, half beef, quarter beef, portion cuts, butcher bag	5.7
	Whole beef, portion cuts	2.9
Method of product marketing (*n* = 35)	Farmer’s market, social media, other marketplace^1^	2.9
	Other marketplace^1^	2.9
	Social media	2.9
	Word of mouth	22.9
	Word of mouth, stand-alone website, farmer’s market, other marketplace^1^	2.9
	Word of mouth, stand-alone website, farmer’s market, social media, other marketplace^1^	5.7
	Word of mouth, stand-alone website, social media, other marketplace^1^	8.6
	Word of mouth, farmer’s market, social media, other marketplace^1^	2.9
	Word of mouth, other marketplace^1^	2.9
	Word of mouth, social media	17.1
	Word of mouth, social media, other marketplace^1^	28.6

^1^Including Shop Kansas Farms & Facebook Marketplace

In terms of beef products sold, most (48.6%) producers sold beef in forms that offered the most flexibility for marketing and sales, including selling as a whole carcass, side, quarter, portion cuts, or the same with the added option of a butcher bag (Table 5). Relatively few (8.6%) sold beef only as a whole carcass, and fewer (5.7%) reported selling only as carcass halves. Overall, varied methods of selling beef cuts were reported, with no single grouping or option of cuts comprising more than roughly a third of producers surveyed. In terms of marketing methods utilized, the vast majority (91.3%) of producers indicated they marketed using multiple methods. Word of mouth (91.3%) was the most popular, followed by social media (65.8%), other marketplaces (54.5%; such as Shop Kansas Farms and Facebook Marketplace), stand-alone websites (17.2%), and farmers’ markets (14.4%). It is noteworthy that the producers surveyed were in part recruited using a list of producers who had previously utilized the Shop Kansas Farms marketplace, and therefore, this marketing channel may have been overrepresented within the surveyed population. Previous work in Oklahoma showed word-of-mouth marketing and the use of social media have been highly effective and have resulted in increased sales of local beef for producers (Langusch, 2021). Overall, these results indicate a variety of marketing methods were utilized by producers to help attract individual customers to their B2C beef offerings.

As the prevalence of B2C beef sales has increased, there is an increased likelihood of negative consumer interactions and complaints to producers. Table 6 outlines producer responses related to negative consumer interactions, complaints, and concerns. Of the producers surveyed, almost 40% (38.9%) reported a consumer complaint/ trouble. Of these, 42.6% reported the customer had problems with the product yield or take-home weight from the animal. Product price or extra charges (such as processing costs) were cited as the reason for the complaints by 49.8% of producers. Only 7.1% of producers indicated customers’ complaints were about product quality. A total of 42.6% of producers included more than one reason for previous customer complaints. Of the 14.3% of producers who indicated “other” consumer complaints, the most commonly identified troubles were associated with order cancelations, lack of knowledge of the process, workmanship, and inspection status. When faced with a complaint, 92.9% of producers indicated they worked to try and resolve the issue, with providing customer education or explanation, and providing a discount as the most common attempts made. Though most producers attempted to resolve the issues, only 75% reported that their attempt resulted in satisfaction for their customers. This number of complaints associated with various parts of the B2C beef process coupled with the number of customers who ended their process unsatisfied (25% of those with complaints) highlights the need for increased education, communication, and resources for those involved with B2C marketing of beef.

**Table 6.** Summary of responses from business-to-consumer (B2C) beef producers regarding complaints or concerns (*N* = 41)

Characteristic	Response	Percentage of responses
Experienced trouble (*n* = 36)	Yes	38.9
	No	61.1
Concern or complaint^1^ (*n* = 14)	Unsatisfied portions	7.1
	Unsatisfied portions, other	7.1
	Low take-home weight	7.1
	Low take-home weight, unsatisfied portions	7.1
	Low take-home weight, unsatisfied portions, high price	7.1
	Low take-home weight, unsatisfied portions, high price, other	7.1
	Low take-home weight, unsatisfied portions, unexpected costs2	7.1
	Low take-home weight, unexpected costs2	7.1
	Unsatisfied quality	7.1
	High price	14.3
	High price, unexpected costs^2^	7.1
	Other	14.3
Other concern or complaint^1^ (*n* = 5)	Lack of knowledge regarding buying process	20.0
	Order cancelations	40.0
	Poor workmanship	20.0
	Customer did not believe product met USDA inspection standards	20.0
Attempted to resolve issue^1^ (*n* = 14)	Yes	92.9
	No	7.1
Description of attempt made^3^ (*n* = 12)	Provided a discount	25.0
	Provided additional beef or other products	8.3
	Other	66.7
Other attempts made^3^ (*n* = 6)	Provided an explanation or education	100.0
Consumer satisfaction with attempt made^3^ (*n* = 12)	Yes	75.0
	No	25.0

^1^Question appeared only to producers who responded yes to experiencing trouble.

^2^Including processing fees and disposal fees.

^3^Question appeared only to producers who responded yes to attempted to resolve issue.

When asked to rate the perceived effectiveness of resources to improve customer experience and prevent future complaints, producers reported widely different opinions related to effectiveness (Table 7). Most of the producers indicated improved consumer knowledge (79.5%) and processor knowledge (65.7%) would be either very or extremely effective at preventing complaints as compared to only 59.4% who indicated improved producer knowledge would be at least very effective. A large majority (78.8%) of producers indicated they thought improved communication between consumers and producers would be at least very effective in preventing future complaints, with 69.7% indicating improved processor and consumer communication would be as effective. In terms of resources to improve customer knowledge, only 31.3% to 45.2% of producers indicated they thought prepared resources provided by state extension sources, USDA or KDA, or non-government sources would be at least very effective, with more than 20% of producers classifying resources from such sources as either only slightly or not effective at all. Producers were much more positive about the impact of customer testimonials, with >77% of producers indicating they thought such resources would be very or extremely effective for improving consumer knowledge. The exact same trends were observed when asked about educational resources to improve processor and producer knowledge, with educational resources from government and non-government entities viewed as very effective or more by only 51% to 56% of producers and testimonials from customers viewed as very or extremely effective by 77% of producers. These results are insightful to the way beef producers who participate in B2C marketing both receive information as well as how they believe their customers receive similar information. Testimonials from customers who have gone through the B2C process were widely viewed more favorably than resources prepared by outside entities to help educate both producers and consumers. Efforts to create such resources to help bridge the knowledge gap should highlight customers’ previous experiences to be most impactful for future customer/producer interactions and sales in the B2C format.

**Table 7.** Summary of responses from business-to-consumer (B2C) beef producers regarding complaints or concerns regarding options to prevent future complaints and concerns (*N* = 41)

Characteristic	Response	Percentage of responses
Effectiveness of improved consumer knowledge (*n* = 34)	Extremely effective	32.4
	Very effective	47.1
	Moderately effective	20.6
	Slightly effective	0.0
	Not effective at all	0.0
Effectiveness of improved producer knowledge (*n* = 32)	Extremely effective	31.3
	Very effective	28.1
	Moderately effective	25.0
	Slightly effective	12.5
	Not effective at all	3.1
Effectiveness of improved locker knowledge (*n* = 32)	Extremely effective	34.4
	Very effective	31.3
	Moderately effective	21.9
	Slightly effective	6.3
	Not effective at all	6.3
Effectiveness of improved communication between consumers and producers (*n* = 33)	Extremely effective	36.4
	Very effective	42.4
	Moderately effective	21.2
	Slightly effective	0.0
	Not effective at all	0.0
Effectiveness of improved communication between consumers and lockers (*n* = 33)	Extremely effective	36.4
	Very effective	33.3
	Moderately effective	18.2
	Slightly effective	12.1
	Not effective at all	0.0
Effectiveness of increased state extension resources to improve consumer knowledge^1^ (*n* = 32)	Extremely effective	12.5
	Very effective	18.8
	Moderately effective	46.9
	Slightly effective	12.5
	Not effective at all	9.4
Effectiveness of increased USDA^2^ or KDA^3^ resources to improve consumer knowledge^1^ (*n* = 31)	Extremely effective	12.9
	Very effective	25.8
	Moderately effective	38.7
	Slightly effective	16.1
	Not effective at all	6.5
Effectiveness of non-government advocates to improve consumer knowledge^1^^,^^4^ (*n* = 31)	Extremely effective	12.9
	Very effective	32.3
	Moderately effective	35.5
	Slightly effective	12.9
	Not effective at all	6.5
Effectiveness of consumer testimonials to improve consumer knowledge^1^ (*n* = 31)	Extremely effective	45.2
	Very effective	32.3
	Moderately effective	19.4
	Slightly effective	0.0
	Not effective at all	3.2
Effectiveness of producer or locker testimonials to improve consumer knowledge^1^ (*n* = 31)	Extremely effective	29.0
	Very effective	29.0
	Moderately effective	25.8
	Slightly effective	12.9
	Not effective at all	3.2
Effectiveness of increased state extension resources to improve producer and locker knowledge^5^ (*n* = 27)	Extremely effective	18.5
	Very effective	33.3
	Moderately effective	22.2
	Slightly effective	22.2
	Not effective at all	3.7
Effectiveness of increased USDA^2^ or KDA^3^ resources to improve producer and locker knowledge^5^ (*n* = 27)	Extremely effective	14.8
	Very effective	40.7
	Moderately effective	25.9
	Slightly effective	18.5
	Not effective at all	0.0
Effectiveness of non-government advocates to improve producer and locker knowledge^5^^,^^4^ (*n* = 26)	Extremely effective	15.4
	Very effective	38.5
	Moderately effective	34.6
	Slightly effective	7.7
	Not effective at all	3.8
Effectiveness of consumer testimonials to improve producer and locker knowledge^5^ (*n* = 27)	Extremely effective	25.9
	Very effective	40.7
	Moderately effective	25.9
	Slightly effective	3.7
	Not effective at all	3.7
Effectiveness of producer or locker testimonials to improve producer and locker knowledge^5^ (*n* = 27)	Extremely effective	29.6
	Very effective	29.6
	Moderately effective	37.0
	Slightly effective	3.7
	Not effective at all	0.0

^1^Question appeared only to producers who responded extremely effective and very effective to improved consumer knowledge.

^2^United States Department of Agriculture.

^3^Kansas Department of Agriculture.

^4^Including National Cattlemen’s Beef Association and Kansas Beef Council.

^5^Question appeared only to producers who responded extremely effective and very effective to improved producer knowledge and improved locker knowledge.

As previously discussed, the COVID-19 pandemic resulted in large interest in B2C beef marketing, with increased interest in B2C marketing across multiple Midwestern states (Langusch, 2021). A large number of producers in the current study reported the short amount of time they had been selling B2C beef, due in part to increased demands from the pandemic. Table 8 outlines producer responses to questions related to their businesses post-2020 to gauge the change the pandemic may have had. The majority (61%) of producers indicated sales directly to individual customers had increased since 2020, with most (75% to 87%) reporting sales to large beef packers, foodservice, and supermarkets were about the same. More than a third (35%) of producers indicated that they first started selling beef in a B2C format in response to increased beef sales to consumers following 2020. An additional 30% of producers indicated they increased the amount of beef they produced to help meet the increased demand for direct-marketed beef following 2020.

**Table 8.** Summary of responses from business-to-consumer (B2C) beef producers regarding estimation of changes in sales in 2020 compared to previous years (*N* = 41)

Characteristic	Response	Percentage of responses
Change of sales to individual consumers (*n* = 41)	Increased	61.0
	About the same	36.6
	Decreased	2.4
Change of sales to foodservice (*n* = 31)	Increased	6.5
	About the same	87.1
	Decreased	6.5
Change of sales to large beef processors (*n* = 32)	Increased	15.6
	About the same	75.0
	Decreased	9.4
Change of sales to supermarkets (*n* = 28)	Increased	7.1
	About the same	85.7
	Decreased	7.1
Others^1^ (*n* = 21)	Increased	0.0
	About the same	100.0
	Decreased	0.0
Responses to increase in sales to individual consumers and foodservice^2^ (*n* = 20)	Began marketing in the B2C^3^ supply chain	35.0
	Consistent pricing	5.0
	Consistent supply	15.0
	Increased demand for beef	10.0
	Increased variety of cuts available	5.0
	Produced a greater amount of beef	30.0

^1^Text responses can be found in Table 2

^2^Question appeared only to producers who responded an increase in individual consumer and foodservice sales in 2020.

^3^Business-to-consumer (**B2C**) is a business model in which consumers purchase products directly from a business.

Finally, Table 9 shows details regarding the business operation of the surveyed B2C beef producers. More than 78% of producers indicated they were either very or extremely interested in increasing sales of beef in the B2C format, with only 3.1% indicating they were not interested at all. In terms of finances, almost all producers (90.6%) indicated they tracked their finances using either a ledger with manual calculation or using a tax software, with 84.4% being somewhat or very satisfied with using these methods. Close to 20% of producers indicated they were at least somewhat dissatisfied with their ability to detect financial opportunities and challenges within their business, but 61.3% were satisfied with their ability to maximize their profit potential. Only one beef producer (3.3%) utilized a benchmarking service to compare the performance of their operations, although all who did not use the benchmarking service reported they would use such services if they were developed. These results underscore the importance of providing assistance to B2C beef producers in relation to their business model and profitability. Currently, no such benchmarking services exist for B2C beef producers as they do for other agricultural commodities. Our results would indicate that if such services were created, they would potentially be highly utilized and relied upon by this group of producers.

**Table 9.** Summary of responses from business-to-consumer (B2C) beef producers regarding business operations (*N* = 41)

Characteristic	Response	Percentage of responses
Level of interest in increased beef sales to individual consumers and foodservice (*n* = 32)	Extremely interested	40.6
	Very interested	37.5
	Moderately interested	18.8
	Slightly interested	0.0
	Not at all interested	3.1
Financial tracking method (*n* = 32)	Ledger with manual calculation	40.6
	Hire an accountant	9.4
	Tax software	50.0
Satisfaction with ease of recordkeeping (*n* = 32)	Very satisfied	40.6
	Somewhat satisfied	43.8
	Neither satisfied nor dissatisfied	6.3
	Somewhat dissatisfied	9.4
	Very dissatisfied	0.0
Satisfaction with detection of financial opportunities and challenges (*n* = 31)	Very satisfied	19.4
	Somewhat satisfied	35.5
	Neither satisfied nor dissatisfied	25.8
	Somewhat dissatisfied	16.1
	Very dissatisfied	3.2
Satisfaction with maximizing profit potential (*n* = 31)	Very satisfied	25.8
	Somewhat satisfied	35.5
	Neither satisfied nor dissatisfied	29.0
	Somewhat dissatisfied	6.5
	Very dissatisfied	3.2
Utilization of benchmarking services to compare operation performance (*n* = 30)	Yes	3.3
	No	96.7
Benchmark service used (*n* = 1)	Kansas farm management	100.0
Accounting basis used (*n* = 27)	Accrual adjusted basis	3.7
	Accrual basis	3.7
	Cash basis	92.6
Interest in using resources, once developed (*n* = 32)	Yes	96.9
	No	3.1

### Consumer Survey

A separate survey was conducted in order to assess consumer perceptions about the B2C beef marketing process. In total, 174 consumers participated, of which 93.1% indicated they had previously purchased beef directly from beef producers. Consumers who indicated they had previously purchased beef were provided one set of questions, while consumers who indicated they had yet to purchase beef were directed to a different set of questions. Of the consumers who had previously purchased beef from producers, the majority (67.8%) reported purchasing other animal products in addition to beef, with a total of 10 different non-beef meats and animal products reported (Table 10). Of the consumers, 52.9% reported purchasing beef in a B2C format only 1 to 5 times, while 21.9% had purchased beef directly from the producers >10 times (Table 11). Most (63.1%) of the consumers indicated they had purchased beef in a B2C format for a period of <5 yr, providing further evidence of the growth and interest in this form of beef marketing during and following the COVID-19 pandemic. Of particular note, 79.4% of consumers identified their knowledge level of the purchasing process as less than or equal to moderately knowledgeable, indicating that though they were participating in the market, they felt like there was more to know and understand than what they felt at their current level. Moreover, the vast majority (96.2%) of consumers indicated they had only purchased products from 1 to 5 different producers, providing additional evidence of the high level of repeat purchases through the same customer/producer relationship that was highlighted in the producer survey and provides further evidence of the level of trust that consumers gain through their relationships with local producers (Telligman et al., 2017).

**Table 10.** Summary of consumer responses regarding purchase of animal products from a local rancher or locker (*N* = 174)

Characteristic	Response	Percentage of responses
Product (*n* = 174)	Beef	32.2
	Beef, chicken or turkey	8.6
	Beef, goat meat	0.6
	Beef, lamb	0.6
	Beef, other	1.7
	Beef, pork	26.4
	Beef, pork, chicken or turkey	16.7
	Beef, pork, chicken or turkey, other	1.1
	Beef, pork, goat meat, lamb, chicken or turkey	0.6
	Beef, pork, lamb	1.1
	Beef, pork, lamb, chicken or turkey	1.7
	Beef, pork, other	1.7
	Chicken or turkey	1.7
	Other	4.0
	Pork	0.6
	Pork, chicken or turkey	0.6
Other responses (*n* = 13)	Bison	7.7
	Cheese	7.7
	Eggs	23.1
	Eggs, honey	7.7
	Yak, water buffalo	7.7
	Have not purchased yet	46.2

**Table 11.** Summary of responses from consumers who previously purchased beef in a business-to consumer (**B2C**)^1^ format (*N* = 174)

Characteristic	Response	Percentage of responses
Frequency of purchase (*n* = 174)	0 times	8.0
	1 to 5 times	52.9
	6 to 10 times	17.2
	11 to 15 times	6.3
	16 to 20 times	2.9
	21 to 25 times	1.1
	26 to 30 times	6.3
	31 to 35 times	0.6
	36 to 40 times	0.6
	41 to 45 times	0.6
	46 to 50 times	1.1
	>100 times	2.3
Years of purchase (*n* = 160)	1 to 5	63.1
	6 to 10	6.9
	11 to 15	10.6
	16 to 20	3.1
	21 to 25	3.8
	26 to 30	3.1
	31 to 35	2.5
	36 to 40	1.3
	41 to 45	3.1
	46 to 50	1.3
	>50	1.3
Familiarity with purchasing (*n* = 160)	Extremely knowledgeable	5.0
	Very knowledgeable	15.6
	Moderately knowledgeable	43.8
	Slightly knowledgeable	27.5
	Not knowledgeable at all	8.1
Number of producers purchased from (*n* = 159)	1 to 5	96.2
	6 to 10	3.1
	>10	0.6
Form of purchase (*n* = 159)	“Butcher bag” program	1.3
	Half beef	15.1
	Half beef, “butcher bag” program	0.6
	Half beef, portion cuts	2.5
	Half beef, quarter beef	7.5
	Half beef, quarter beef, portion cuts	2.5
	Half beef, quarter beef, portion cuts, “butcher bag” program	1.3
	Portion cuts	24.5
	Portion cuts, “butcher bag” program	3.1
	Quarter beef	17.0
	Quarter beef, portion cuts	8.2
	Quarter beef, portion cuts, “butcher bag”	1.3
	Whole beef	3.8
	Whole beef, half beef	2.5
	Whole beef, half beef, portion cuts	3.8
	Whole beef, half beef, portion cuts, “butcher bag” program	0.6
	Whole beef, half beef, quarter beef	0.6
	Whole beef, half beef, quarter beef, portion cuts	1.3
	Whole beef, portion cuts	0.6
	Whole beef, quarter beef	1.3
	Whole beef, quarter beef, portion cuts	0.6
Self-characterization of beef purchasing (*n* = 153)	100% local beef	35.9
	75% local beef	37.3
	50% local beef	11.1
	25% local beef	15.7

^1^Business-to-consumer is a business model in which consumers purchase products directly from a business, such as meat/livestock for slaughter directly from livestock producers.

The forms of beef purchased by consumers varied widely and were reflective of the producers' survey responses (Table 11). The most popular form of purchase was portion cuts, reported by 47.2% of customers, with beef sides (29.5%), quarters (26.5%), and whole carcasses (8.8%) also purchased. These data seem to indicate that customers preferred the ability to purchase fewer pounds of product in the form of cuts, quarters, and sides compared to whole carcasses. Whole beef carcasses typically produce a relatively large quantity of product for customers, with a typical 363 kg carcass producing 202 kg of retail product (McKillip et al., 2018). Many customers may not have the freezer storage space to store such an amount, and therefore have sought out opportunities to purchase lower quantities of beef, and thus portion cuts or quarters may better fit their needs.

The majority (73.2%) of consumers indicated that more than 75% of the beef they purchase is through local B2C beef producers. Of the consumers who indicated they had previously purchased beef in a B2C format, 63.1% indicated it was their first purchase (Table 12). Consumers reported a varied number of ways that they were able to locate producers, including by personal recommendation (36.2%), through social media (23.9%), other marketplaces (28.5%), and through stand-alone websites (2.5%). These are very similar to the marketing channels identified by producers, with word-of-mouth marketing being reflected in the recommendation response of consumers. Social media was also a very common channel, and one that producers had indicated putting efforts into as well. Again, the other marketplace included Shop Kansas Farms, which was used to help recruit consumers for the survey and thus would be expected to be represented in the data, with the possibility of this estimate being inflated. Of the other methods identified, most (66.7%) consumers reported a personal relationship with the producer for beef purchases.

**Table 12.** Summary of responses from consumers who previously purchased beef in a business-to-consumer (**B2C**)^1^ format regarding most recent purchase (*N* = 174)

Characteristic	Response	Percentage of responses
Most recent purchase was first purchase (*n* = 157)	Yes	63.1
	No	36.9
Method of finding product (*n* = 154)	Stand-alone website	0.6
	Stand-alone website, other marketplace^2^	0.6
	Stand-alone website, social media	1.3
	Farmer’s market	3.2
	Farmer’s market, social media	0.6
	Farmer’s market, social media, other marketplace^2^	0.6
	Other marketplace^2^	23.4
	Other	11.7
	Recommendations	31.8
	Recommendations, farmer’s market	0.6
	Recommendations, other marketplace^2^	2.6
	Recommendations, other	0.6
	Recommendations, social media	0.6
	Social media	15.6
	Social media, other marketplace^2^	3.9
	Social media, other	1.9
Other method of finding product (*n* = 18)	4-H animal	5.6
	Personal relationship	66.7
	Local locker/butcher	22.2
	KC Food Circle	5.6

^1^Business-to-consumer is a business model in which consumers purchase products directly from a business, such as meat/livestock for slaughter directly from livestock producers.

^2^Including Shop Kansas Farms & Facebook Marketplace.

A high percentage (94.3%) of consumers indicated that they had never experienced trouble or had a complaint regarding their beef purchase (Table 13). Of those that did, reasons were varied and included issues related to take-home weight, product quality, portion size, tenderness, and processor issues. Of the consumers who identified low take-home weights, 66.7% reported not requesting organ meat and requesting boneless products, both of which would reduce the total amount of product yield from an animal, with bone and fat weight typically representing between 20% and 22% of the overall carcass weight and ordering all cuts boneless typically resulting in an additional 2% of carcass weight lost through discarded bones (McKillip et al., 2018). Interestingly, only 44.4% of customers who said they had previously experienced an issue reported the producer attempted to resolve the issue, which is a much lower rate than what was identified in the producer survey. Of those who did attempt to help, strategies included providing additional beef or providing an explanation/education for the customer, with 75% of consumers reporting they were satisfied with the producer’s response. This is reflected in the 100% of consumers who reported that despite the trouble, they intended to continue to purchase B2C beef due to multiple reasons including they were overall pleased with the product quality, wanted to support the local economy, or intended to switch producers.

**Table 13.** Summary of responses from consumers who previously purchased beef in a business-to-consumer (**B2C**)^1^ format regarding complaints or concerns (*N* = 174)

Characteristic	Response	Percentage of responses
Experienced trouble (*n* = 157)	Yes	5.7
	No	94.3
Concern or complaint^2^ (*n* = 9)	Unsatisfied portions	11.1
	Low take-home weight, other	11.1
	Low take-home weight, unsatisfied portions	11.1
	Unsatisfied quality, unsatisfied portions, other	11.1
	Unsatisfied quality, unsatisfied portions, limited variety, other	11.1
	Unsatisfied quality, low take-home weight, unsatisfied portions, other	11.1
	Limited variety	11.1
	Other	22.2
Other concern or complaint^2^ (*n* = 6)	Lack of knowledge regarding cut order	33.3
	Poor workmanship	16.7
	Bad experience	16.7
	Tough meat	16.7
	Issue with locker	16.7
Requested bones and organs^3^ (*n* = 9)	Yes	33.3
	No	66.7
Requested boneless steaks^3^ (*n* = 9)	Yes	66.7
	No	33.3
Rancher or locker attempted to resolve issue^2^ (*n* = 9)	Yes	44.4
	No	55.6
Description of attempt made^4^ (*n* = 4)	Provided additional beef or other products	50.0
	Provided an explanation	50.0
Consumer satisfaction with attempt made^4^ (*n* = 4)	Extremely satisfied	50.0
	Somewhat satisfied	25.0
	Neither satisfied nor dissatisfied	25.0
Intention to continue purchasing B2C beef (*n* = 9)	Yes	100.0
Reason for continued purchase^5^ (*n* = 7)	Pleased with quality	42.9
	Intend to switch producer/locker	28.6
	Support local economy	14.3
	Access to specialty cuts	14.3

^1^Business-to-consumer is a business model in which consumers purchase products directly from a business, such as meat/livestock for slaughter directly from livestock producers.

^2^Question appeared only to consumers who responded yes to experiencing trouble.

^3^Question appeared only to consumers who responded low take-home weight to concern or complaint.

^4^Question appeared only to consumers who responded yes to attempted to resolve issue.

^5^Question appeared only to consumers who responded yes to intention to continue purchasing.

Of the consumers who identified not purchasing local beef yet (Table 14), most (91.7%) reported they probably or definitely would. When asked about the barriers to purchasing beef in the B2C format, cost was identified by 24.9% of consumers. This is in-line with previous studies that have identified the high up-front cost as a major barrier for consumers to enter the B2C market (Langusch, 2021). Moreover, a lack of freezer space or product quantity was identified by 66.7% of consumers as the major barrier. This is in agreement with the responses of customers who had purchased B2C beef and identified purchasing portion cuts at a greater rate than whole carcasses or carcass halves. The ability for producers to offer smaller portions in the form of portion cuts or beef quarters may offer an opportunity for greater sales and new customers if the storage quantity demands of B2C beef can be reduced through innovative marketing strategies by producers.

**Table 14:** Summary of responses from consumers who have no experience purchasing beef in a business-to-consumer (**B2C**)^1^ format (*N* = 174)

Characteristic	Response	Percentage of responses
Considered purchasing beef in B2C format (*n* = 12)	Definitely yes	66.7
	Probably yes	25.0
	Might or might not	0.0
	Probably not	8.3
	Definitely not	0.0
Barriers to purchasing beef in B2C format^2^ (*n* = 12)	Not cost effective, other	8.3
	Not cost effective, quantity too large, lack of freezer space	8.3
	Lack of freezer space	25.0
	More expensive than retail beef	8.3
	Quantity too large	16.7
	Quantity too large, lack of freezer space	8.3
	Quantity too large, lack of freezer space, other	16.7
	Other	8.3
Other barriers to purchasing beef in B2C format^2^ (*n* = 4)	Financial burden	25.0
	Unsure how to order	25.0
	Processor in inconvenient location	25.0
	Unsure on costs	25.0

^1^Business-to-consumer is a business model in which consumers purchase products directly from a business, such as meat/livestock for slaughter directly from livestock producers.

^2^Question appeared only to consumers who responded yes to consider purchasing.

When asked about strategies on how to improve the B2C system and strategies to avoid future complaints and concerns, consumer responses did not differ much from the producer responses (Table 15). Overall, 56% to 62% of consumers identified improved knowledge of consumers, producers, and processors would be either very or extremely effective, though only 61.6% identified improved consumer knowledge would be this beneficial, a much lower percentage than was identified by producers. Improved communication between consumers and beef producers (71.9%) and between consumers and processors (73.8%) were also believed to be either very or extremely effective at preventing future complaints. This was slightly lower for the producer and consumer communication question than what was identified by producers, in which more than 78% felt improved communication between consumers and producers would be at least very effective. This shows a discrepancy between how producers and consumers feel about this relationship, with producers feeling as if communication needs to be better between the two groups, while consumers have lesser concerns about this communication.

**Table 15.** Summary of responses from consumers who previously purchased beef in a business-to-consumer (**B2C**)^1^ format regarding options to prevent future complaints and concerns (*N* = 174)

Characteristic	Response	Percentage of responses
Effectiveness of improved consumer knowledge (*n* = 143)	Extremely effective	21.7
	Very effective	39.9
	Moderately effective	28.7
	Slightly effective	4.2
	Not effective at all	5.6
Effectiveness of improved rancher knowledge (*n* = 139)	Extremely effective	18.0
	Very effective	38.1
	Moderately effective	29.5
	Slightly effective	7.9
	Not effective at all	6.5
Effectiveness of improved locker knowledge (*n* = 140)	Extremely effective	26.4
	Very effective	35.0
	Moderately effective	23.6
	Slightly effective	9.3
	Not effective at all	5.7
Effectiveness of improved communication between consumers and ranchers (*n* = 139)	Extremely effective	25.9
	Very effective	46.0
	Moderately effective	20.1
	Slightly effective	4.3
	Not effective at all	3.6
Effectiveness of improved communication between consumers and lockers (*n* = 145)	Extremely effective	31.7
	Very effective	42.1
	Moderately effective	15.9
	Slightly effective	5.5
	Not effective at all	4.8
Effectiveness of increased state extension resources^2^ (*n* = 82)	Extremely effective	15.9
	Very effective	28.0
	Moderately effective	39.0
	Slightly effective	13.4
	Not effective at all	3.7
Effectiveness of increased USDA^3^ or KDA^4^ resources^2^ (*n* = 80)	Extremely effective	12.5
	Very effective	27.5
	Moderately effective	30.0
	Slightly effective	26.3
	Not effective at all	3.8
Effectiveness of non-government advocates^2^^,^^5^ (*n* = 82)	Extremely effective	17.1
	Very effective	24.4
	Moderately effective	37.8
	Slightly effective	18.3
	Not effective at all	2.4
Effectiveness of consumer testimonials^2^ (*n* = 80)	Extremely effective	35.0
	Very effective	40.0
	Moderately effective	17.5
	Slightly effective	3.8
	Not effective at all	3.8
Effectiveness of other suggestions^2^ (*n* = 25)	Extremely effective	12.0
	Very effective	28.0
	Moderately effective	36.0
	Slightly effective	0.0
	Not effective at all	24.0
Other suggestions^2^ (*n* = 19)	Better explanation of cut lists	10.5
	Better explanation of take-home weight	5.3
	Easier to find producers	21.1
	Improve communication	15.8
	Keep prices competitive	10.5
	Provide more background on ranchers	5.3
	Utilize social media for advertising	26.3
	Visit facilities	5.3

^1^Business-to-consumer is a business model in which consumers purchase products directly from a business, such as meat/livestock for slaughter directly from livestock producers.

^2^Question appeared only to consumers who responded extremely effective and very effective to improved consumer knowledge.

^3^United States Department of Agriculture.

^4^Kansas Department of Agriculture.

^5^Including National Cattlemen’s Beef Association and Kansas Beef Council.

Interestingly, consumers had similar feelings about the effectiveness of resources generated through government and non-government resources as producers (Table 15). Less than 44% of consumers felt additional resources from state extension sources, the USDA or KDA, or non-governmental sources would be either very or extremely effective. More than 30% of consumers identified resources from USDA and KDA as either only slightly effective or not effective at all. But, similar to producers, personal testimonials were viewed very favorably. 75% of consumers thought additional consumer testimonials would be either very or extremely effective at preventing future issues. The strong agreement between both producer and consumer responses related to the need for greater numbers of consumer testimonials and the perceived value of these in preventing future issues is noteworthy. Such consumer testimonials have been shown to be highly effective marketing tools, but can negatively influence sales if the consumer reviews appear to be exaggerated (Shimp et al., 2007). Nonetheless, efforts to improve the B2C model and the relationship between consumers and producers in marketing beef through this channel should focus on consumer testimonials and the individual customer’s experience through all aspects of the process from identification of producers, understanding the processing of the animal, through end-product yield and storage. Such testimonials would provide a valuable link between consumers and producers that can help build understanding and trust and ultimately provide a successful foundation for B2C beef marketing.

## Conclusion

Interest from both consumers and producers in B2C marketing of beef has increased in recent years. A lack of a full understanding of the process by consumers coupled with producers not fully understanding how to enter and grow within this market has created unique demands on the system. Data from the current work underscores the importance of continued and improved communication between producers and consumers for success, with needs for educational materials to help eliminate potential problems. Both consumers and processors identified personal testimonials among the most impactful, and future educational efforts should focus on their inclusion. Moreover, the large amount of beef from a single animal and the required storage space were viewed as barriers for new consumers to participate in B2C marketing. Thus, producers should increase their ability to market smaller portions and lower volumes of cuts, such as the sale of portion cuts as opposed to sides or whole carcasses, in order to increase consumer ease and willingness to participate in the B2C model. Business-to-consumer marketing of beef offers both consumers and producers the ability to participate in the local food system. Therefore, efforts are needed and should continually be made in order to help facilitate positive interactions for such B2C beef sales.
